# Both respiration and photosynthesis determine the scaling of plankton metabolism in the oligotrophic ocean

**DOI:** 10.1038/ncomms7961

**Published:** 2015-04-24

**Authors:** Pablo Serret, Carol Robinson, María Aranguren-Gassis, Enma Elena García-Martín, Niki Gist, Vassilis Kitidis, José Lozano, John Stephens, Carolyn Harris, Rob Thomas

**Affiliations:** 1 Departamento de Ecología y Biología animal, Universidad de Vigo, E36310 Vigo, Spain; 2 Estación de Ciencias Marinas de Toralla, Universidad de Vigo, Toralla island, E-36331 Vigo, Spain; 3 School of Environmental Sciences, University of East Anglia, Norwich Research Park, Norwich NR4 7TJ, UK; 4 WK Kellogg Biological Station, Michigan State University, 3700 E. Gull Lake Drive, Hickory Corners MI 49060, USA; 5 Plymouth Marine Laboratory, Prospect Place, Plymouth PL1 3DH, UK; 6 British Oceanographic Data Centre, Joseph Proudman Building, 6 Brownlow Street, Liverpool L3 5DA, UK

## Abstract

Despite its importance to ocean–climate interactions, the metabolic state of the oligotrophic ocean has remained controversial for >15 years. Positions in the debate are that it is either hetero- or autotrophic, which suggests either substantial unaccounted for organic matter inputs, or that all available photosynthesis (P) estimations (including ^14^C) are biased. Here we show the existence of systematic differences in the metabolic state of the North (heterotrophic) and South (autotrophic) Atlantic oligotrophic gyres, resulting from differences in both P and respiration (R). The oligotrophic ocean is neither auto- nor heterotrophic, but functionally diverse. Our results show that the scaling of plankton metabolism by generalized P:R relationships that has sustained the debate is biased, and indicate that the variability of R, and not only of P, needs to be considered in regional estimations of the ocean's metabolic state.

## Abstract (web summary)

Whether the oligotrophic ocean is net heterotrophic or net autotrophic has been a matter of debate for many years. Here Serret *et al*. show that, rather than being one or the other, the oligotrophic ocean is functionally diverse, with different metabolic states in different gyres.

## Graphical abstract

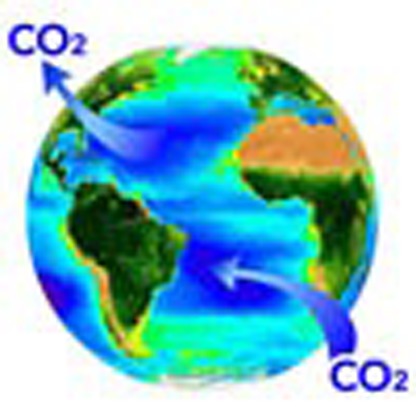

The microbial biota of the surface ocean is a prime determinant of the ocean's productivity and biogeochemical functioning. Marine phytoplankton is responsible for half of the Earth's oxygenic photosynthesis, which contributes to the redox state of the planet and the global cycles of C and O_2_ in the ocean and atmosphere, affecting the global climate and creating habitable conditions for many organisms^1^,^2^. Part of the photosynthetically produced organic matter is respired in the surface ocean, either to support the maintenance costs of the phytoplankton or by the heterotrophic activity of other microbes. This oxidation counterbalances the fluxes of C and O_2_ from photosynthesis, and reduces the net amount of organic matter available for consumption by metazoans. The difference between plankton photosynthesis (P) and auto- plus heterotrophic respiration (R) is net community production (NCP), the amount of photosynthetically fixed C available for sequestration to the deep ocean or for transfer up the marine food web^3^. Over significant scales, NCP summarizes the energy flow and metabolic state of a planktonic ecosystem, setting upper bounds to its contribution to O_2_ and CO_2_ fluxes, and hence to global climate.

The oligotrophic ocean is the most extensive biome of the world, occupying about half of the Earth's surface. Its areal productivity is limited because of the strong vertical stratification of the water column that reduces nutrient inputs to the illuminated zone. However, its extent makes its contribution to global productivity, C export and biologically driven ocean–atmosphere fluxes significant^4^,^5^,^6^. In addition, climate change is increasing both the area^7^ and stratification of oligotrophic gyres^7^,^8^, with feedbacks to the biological rates and the metabolic state. Despite its importance, the metabolic state of the oligotrophic ocean has remained controversial^3^, since del Giorgio *et al*.^9^ published a seminal paper on the relationship between primary production and bacterial respiration in the ocean. Direct measurements of community metabolism in low-production ecosystems show a prevalence of heterotrophy (P<R)^10^, that leads to the ‘intriguing, if not disquieting' idea that the extensive and remote oligotrophic ocean is heterotrophic^3^. These results are based on the *in vitro* incubation of small seawater samples (∼125 ml) during short periods of time (∼24 h), which are susceptible to biases from the bottle confinement of natural plankton communities^11^. An alternative to incubation-based methods is to study the indirect impact of plankton metabolism on inventories of biogenic or limiting compounds in large water bodies (km^2^) during long periods of time (weeks to months)^12^,^13^,^14^. Contrary to direct measurements of planktonic activity, results from this approach show a prevalence of autotrophy (P>R) in the oligotrophic ocean^11^. These calculations involve a large uncertainty derived from the estimations of water mass mixing and air–sea gas exchange^10^, and assume a constancy in gradients of stocks and water transport that conflicts with non-steady ocean dynamics^15^.

Beyond methodological biases that have played a major role in the debate^3^,^10^,^11^, these two approaches measure different processes over completely different time and spatial scales—snapshots of the NCP of single communities versus the integrative metabolism of a succession of communities in a water mass. Comparison hence requires scaling-up *in vitro* data to the large and long spatial and temporal extent of *in situ* estimates. To this aim, empirical P:R relationships have been used to predict NCP from the extensive global data set of P (mainly ^14^C derived)^16^,^17^,^18^,^19^. All the extrapolations carried out during the last 15 years have found heterotrophy to prevail whenever P is low, leading some authors to conclude that the oligotrophic ocean is heterotrophic and the (autotrophic) *in situ* data are biased^10^. According to these authors, the input of organic matter to the open ocean is much higher than the observations of stocks and export from the euphotic zone, and non-oxygenic autotrophic processes (not included in P estimations) are significantly more relevant than currently believed^10^. On the contrary, others have concluded that the oligotrophic ocean is autotrophic, and that all the *in vitro* measurements of plankton production—including tens of thousands of ^14^C measurements—could be biased because of the perturbation of the light conditions^11^. Such a widespread and constant state of autotrophy would require a supply of inorganic nutrients to the surface oligotrophic ocean at rates much higher than observations of stocks and fluxes indicate^15^. Both positions hence ultimately imply unresolved explanatory hypotheses that would force a reconsideration of our knowledge about the magnitude, variability and control of organic matter production and cycling in the open ocean.

Despite the lengthy discussion on methodological biases, very little attention has been paid to the scaling procedure that is essential for data comparisons. In all cases, the empirical P:R^16^,^17^,^18^,^19^ or P/R:P^17^,^20^ relationships are assumed universal^16^,^17^,^18^,^20^ or constant for a certain latitudinal band (for example, 10–40°)^19^, which relies on some important and untested assumptions. The idea that NCP (that is, P minus R) may be predicted in the oligotrophic ocean from P alone is based on the hypothesis that changes in P but not in R control the metabolic balance either globally^10^,^16^,^17^,^18^,^20^ or at low latitudes^11^,^19^. This may occur because the variability of R is irrelevant or minor^20^,^21^,^22^, or because it is tightly coupled to the variability of P^16^,^17^,^18^,^19^. In either case, the use of a single P:R relation implies that the influence of P-independent processes on heterotrophic R (for example, magnitude and quality of allochthonous organic matter inputs, consumption of organic matter accumulated during productive phases, composition, structure and activity of the heterotrophic communities) are uniform throughout at least the low latitude band of the global ocean. This is a very tenuous assumption, as even in the most extensive data sets^18^,^19^ used both to calculate balances and to derive P:R relationships^10^,^11^,^18^,^19^, the great majority of data from the oligotrophic ocean come from one province, the Eastern Atlantic gyre (NAST-E). However, a reduced number of observations in the South Atlantic gyre have suggested differences in community metabolism that would reflect distinct P:R relationships^23^,^24^.

To test the assumption that no regional differences exist in either the P:R relationship or the metabolic balance throughout the oligotrophic ocean, that is, to assess the basis of the scaling procedure sustaining the debate, we have compiled 194 vertical profiles (median five depths) in the epipelagic zone (defined here as the layer between the surface and the 1% of photosynthetically active radiation—PAR—depth) of *in vitro* derived P, R and NCP measurements (ΔO_2_ after 24-h light and dark incubations) made across the Atlantic (50° N–50° S) during 10 Atlantic Meridional Transect (AMT11-18, AMT21-22) cruises from 2000 to 2012. All the cruises except AMT17 and AMT18 included both the North and South gyres (NAST and SATL). This is a very consistent data set in terms of sampling, incubation and analytical methodology and precision, it is equivalent to ∼76% of non-AMT Atlantic data in the latest published global NCP data set^18^, and includes 46 profiles from the very undersampled SATL. The analysis of this data set confirms the prevalence of net heterotrophy in the NAST, but shows that the plankton metabolism in the similarly oligotrophic SATL was predominantly autotrophic. Our data reveal significant differences in R, P:R relationship and NCP between the two Atlantic ocean gyres, indicating that the oligotrophic ocean is functionally diverse. This would bias any regional to global prediction of NCP based on generalized P:R relationships, constraining the scaling of plankton metabolism to functionally coherent ecosystems.

## Results

### Plankton metabolic rates in the Atlantic gyres

**Figure 1:**
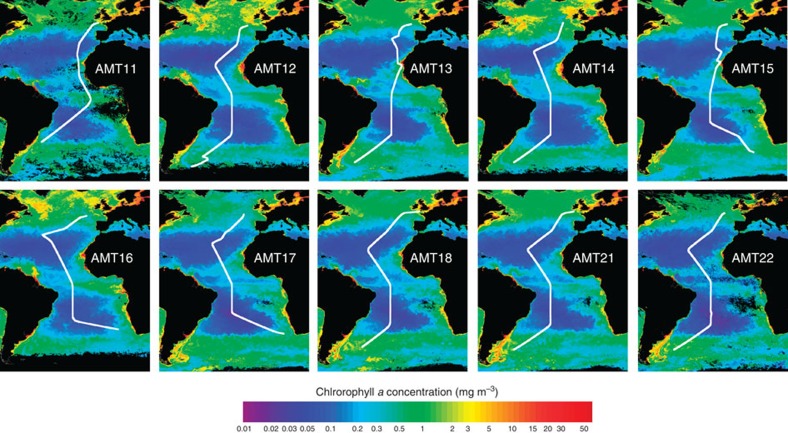
The AMT cruises. Tracks of Atlantic Meridional Transect (AMT) cruises 11–18 and 21–22 overlaid on SeaWiFS chlorophyll *a* composites. After AMT11 (September–October 2000), cruises took place biannually from May 2003 (AMT12) until October 2005 (AMT17); AMT18, 21 and 22 took place in September–October 2008, 2011 and 2012, respectively. Earth observation data produced by the ESA Ocean Colour Climate change Initiative, courtesy of the NERC Earth Observation Data Acquisition and Analysis Service, Plymouth, the Ocean Biology Processing Group, NASA and European Space Agency.

The AMT cruises traversed several biogeographic provinces of the Atlantic Ocean (Fig. 1). Gyre stations were identified when the depth of the thermocline and chlorophyll *a* maxima were >100 m (∼20–38° N for the NAST; ∼10–32° S for the SATL).

**Figure 2:**
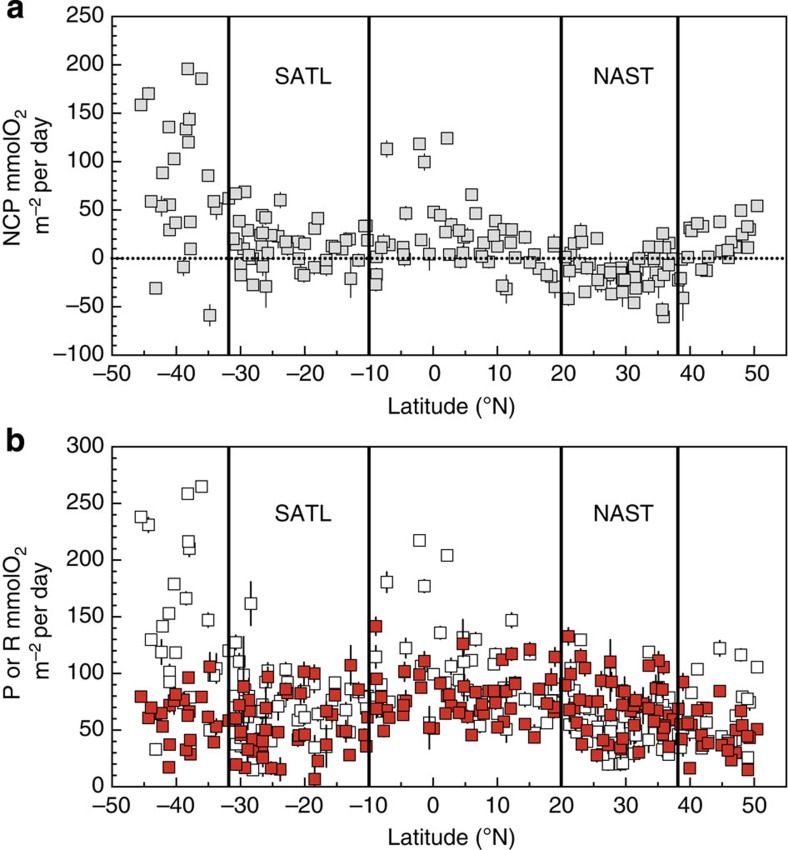
Integrated metabolism in the epipelagic zone. Latitudinal variation of integrated (**a**) NCP and (**b**) photosynthesis (P, open squares) and respiration (R, red filled squares) data during the AMT11 to AMT22 cruises. The error lines represent the propagated s.e. The dotted line on **a** is the NCP=0 line, indicating the transition from auto- to heterotrophy. The approximate location of the South Atlantic gyre (SATL) and North Atlantic gyre (NAST) provinces is indicated.

**Figure 3:**
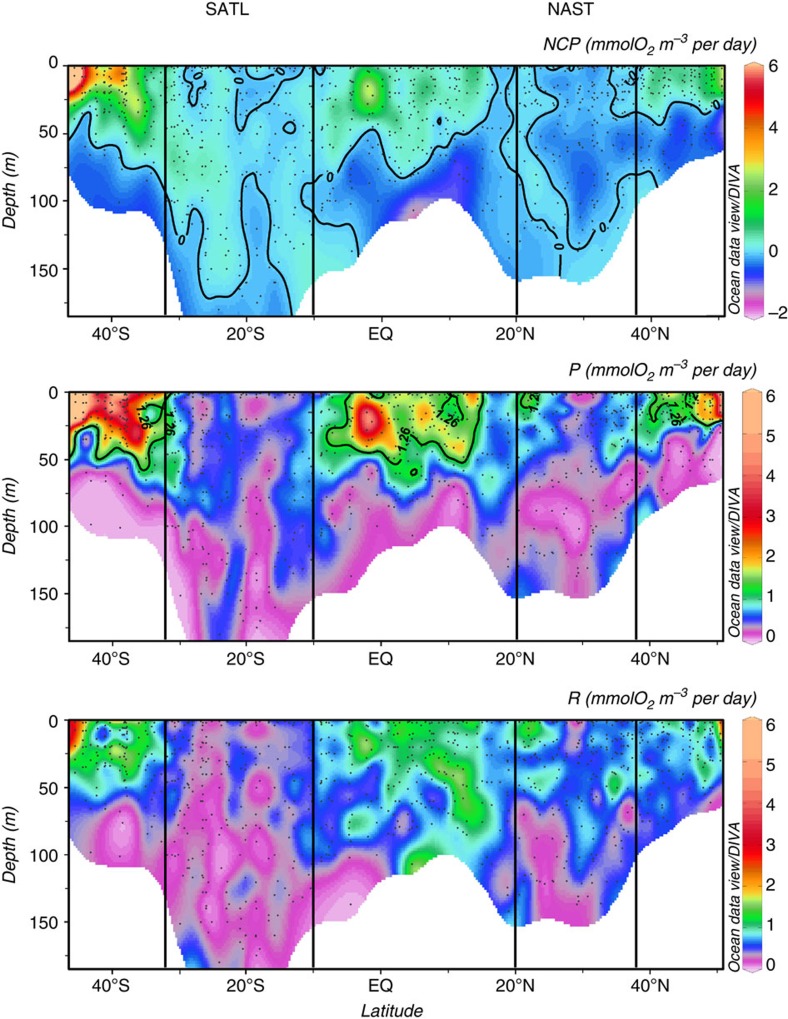
Variation of plankton metabolism through the epipelagic waters of the Atlantic Ocean. Composite depth-latitudinal sections of plankton NCP, P and R (AMT11–22). Data from the NW Africa upwelling from AMT11, AMT13 and AMT15 are excluded. The 0 mmolO_2_ m^−3^ per day NCP isoline marks the auto-heterotrophy transition. The 1.26-mmolO_2_ m^−3^ per day P isoline marks the threshold for net heterotrophy in Regaudie-de-Gioux and Duarte^18^. The approximate location of the SATL and NAST provinces is indicated.

**Figure 4:**
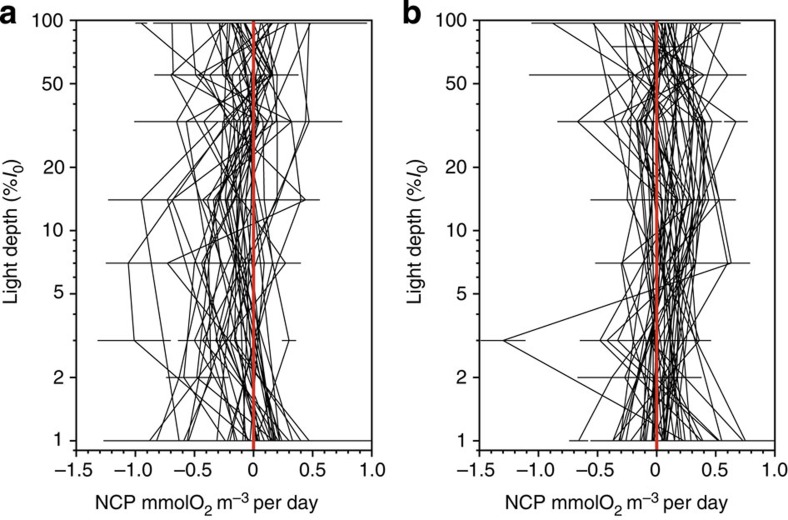
Vertical variation of NCP in the Atlantic gyres. Profiles of NCP measurements (±s.e.m.) at different light depths in the epipelagic zone of the (**a**) NAST and (**b**) SATL stations in the AMT11–22 data set. The red lines mark the 0-mmolO_2_ m^−3^ per day NCP.

**Figure 5:**
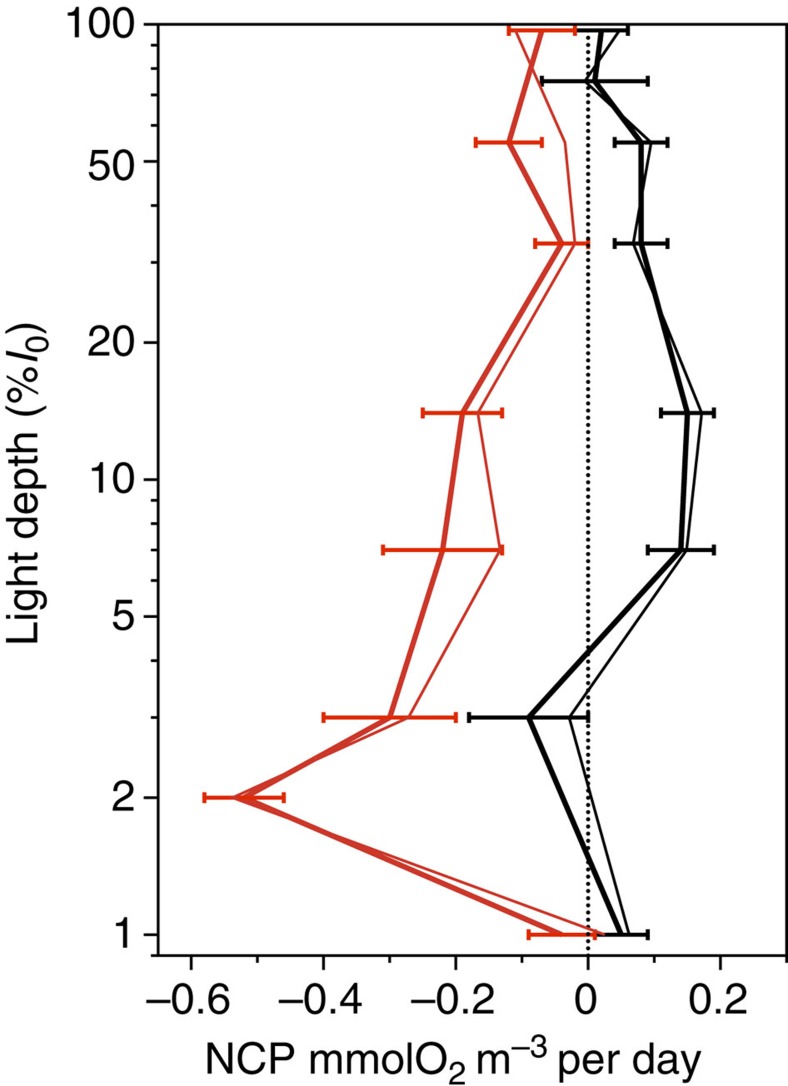
Vertical distribution of NCP in the Atlantic gyres. Profiles of the average (±s.e.m.; thick lines) and median (thin lines) NCP rates at different light depths in the epipelagic zone of the NAST (red lines) and SATL (black lines) stations in the AMT11–22 data set. The dotted line marks the 0-mmolO_2_ m^−3^ per day NCP.

Integrated NCP was clearly different between the NAST and SATL (*t*-test (*n*=91)=−7.14, *P*=0.0001; Fig. 2a, Table 1). Net heterotrophy prevailed in the NAST, agreeing with the literature^10^,^17^,^18^,^19^,^20^,^23^,^24^; 85% of our integrated NCP data were negative, the mean (± s.e.m.) NCP was −14.55±2.42 mmolO_2_ m^−2^ per day and the 95% confidence interval was −19.31 to −9.79 mmolO_2_ m^−2^ per day. However, in the SATL, a balanced or net autotrophic metabolism prevailed (78% of the data), with a mean NCP of 9.78±2.34 mmolO_2_ m^−2^ per day and a 95% confidence interval of 4.96 to 14.59 mmolO_2_ m^−2^ per day. The composite vertical and latitudinal variability of volumetric rates during AMT11 to AMT22 shows that the significant differences in NCP between the NAST and SATL were consistent throughout the entire epipelagic zone (*t*-test (*n*=433)=−6.63, *P*=0.0001; Fig. 3). Sixty-two per cent of the 202 volumetric data from the NAST were net heterotrophic, with a mean (± s.e.m.) of −0.12±0.02 mmolO_2_ m^−3^ per day, while 63% of the 233 data from the SATL were net autotrophic, with a mean (± s.e.m.) of 0.06±0.02 mmolO_2_ m^−3^ per day (Table 1). Although these percentages in the NAST concur with the literature (57% (ref. 10)), conversion of volumetric data into discrete categories with a cutoff NCP of zero (that is, auto- or heterotrophic) misrepresents the prevailing metabolism because of the relatively high variability of these very low rates (Fig. 4), especially in the upper 25 m (∼33% light depth) and at the 1% light depth, where the mean NCP rates were very close to zero at both the NAST and SATL (Fig. 5). This explains the lower percentage of the volumetric data that were auto- or heterotrophic in the SATL and NAST (63 and 62%, respectively), compared with the depth integrated data (78–85%). Vertical profiles of the mean and median NCP through the epipelagic zone provide a more realistic account of the clear differences in the prevailing metabolism of the two gyres (Fig. 5).

Most interestingly, the regional differences observed in NCP were not caused only by differences in P, but fundamentally by the variation of R. The assumed relative constancy of R^16^,^17^,^18^,^19^,^20^,^21^,^22^ is only found when we analyse the entire latitudinal range of the transect, that is, when the highly productive equatorial and temperate provinces are included together with the oligotrophic gyres (Figs 2b and 3). However, within the two gyres, we found similar ranges of variability for both volumetric and integrated P and R rates (Figs 2b and 3 and Table 1), and this variability of R has consequences for the differences in metabolic balance between the gyres. The mean integrated P was lower in the heterotrophic NAST than in the autotrophic SATL (57.51±3.50 versus 68.32±3.13 mmolO_2_ m^−2^ per day, respectively), consistent with the hypothesis that P alone controls NCP^10^,^11^,^12^,^13^,^14^,^15^,^16^,^17^,^18^,^19^,^20^. However, the mean integrated R was lower in the autotrophic SATL than in the heterotrophic NAST (58.53±3.85 versus 72.09±3.47 mmolO_2_ m^−2^ per day), where very few data lower than ∼35 mmolO_2_ m^−2^ per day were found (Fig. 2b). Volumetric rates emphasize the importance of R to the significant differences in NCP between the NAST and SATL. R was significantly higher in the heterotrophic NAST (0.68±0.03 mmolO_2_ m^−3^ d^−1^) than in the autotrophic SATL (0.48±0.02 mmolO_2_ m^−3^ per day; *t*-test (*n*=439)=6.08, *P*=0.0001), while P rates were not significantly different (0.56±0.02 and 0.55±0.02 mmol O_2_ m^−3^ per day in the NAST and SATL, respectively; *t*-test (*n*=433)=0.56, *P*=0.57; Table 1). Volumetric rates of P were mostly lower in both gyres than the latest published threshold P for net heterotrophy (1.26 mmolO_2_ m^−3^ per day)^18^ (Fig. 3). However, the corresponding prediction of net heterotrophy was only fulfilled in the NAST, precisely the oligotrophic province that contributes the most to the global data set whose generalized P:R relationship sustains both this threshold^18^ and the hypothesis of relative R constancy^10^,^18^.

### Scaling plankton metabolism in the Atlantic gyres

**Figure 6:**
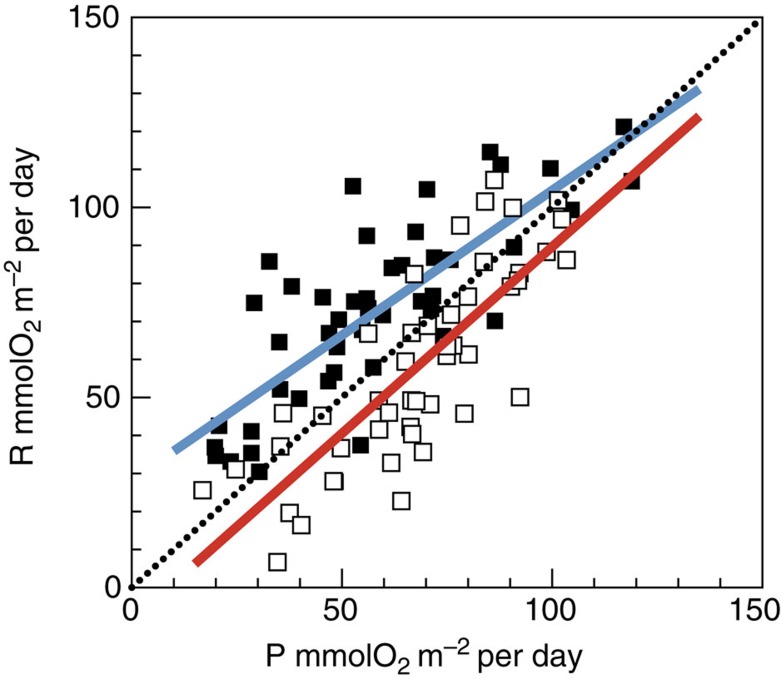
Relationship between P and R. Relationship between integrated rates of photosynthesis (P) and respiration (R) in the NAST (filled squares) and SATL (open squares). The blue line is the regression line for the NAST data: *R*=0.76P+28.25, *R*^2^=0.59, *n*=47. The red line is the regression line for the SATL data: *R*=0.98P−8.56; *R*^2^=0.64; *n*=46. Both the slope and intercept of the two equations are significantly different (*t*-test (*n*=89)=12.89, *P*=0.0001; *t*-test (*n*=89)=22.28, *P*=0.0001, respectively). The dotted line indicates the 1:1 line.

The difference in the relative importance of R, and its consequences for scaling the metabolic balance are clearly shown in a plot of the P:R relationships in the two gyres (Fig. 6). We focus on the integrated rates because they provide a more accurate representation of the interaction of auto- and heterotrophic processes in the epipelagic ocean^16^,^23^. The NAST P:R relationship resembles the generalized relationships that have been used to scale-up *in vitro* data for the last 15 years^16^,^17^,^18^,^19^. The degree of heterotrophy is inversely related to the magnitude of P, so that below a certain threshold P (∼120 mmolO_2_ m^−2^ per day), the net heterotrophy would always be predicted. As P in the oligotrophic ocean is typically below calculated thresholds, using this type of relationship to extrapolate NCP, together with the assumption of trophic invariability, has led to the conclusions that either the oligotrophic ocean is net heterotrophic and requires substantial allochthonous inputs of organic matter^10^, or *in vitro* data are biased^11^. However, the SATL P:R relationship is essentially a 1:1 relationship (*t*-test, *P*<0.01), indicating a tight coupling of local auto and heterotrophic processes. This relationship would never predict net heterotrophy from P alone. Actually, neither the NAST nor SATL relationship is able to correctly predict the metabolic balance in the other oligotrophic gyre from P alone. Consequently, differences in R between the two gyres, and not only in P are critical for the prediction of metabolic balances in the oligotrophic Atlantic, which should therefore be based on distinct P:R relationships for (at least) each of the two gyres.

### Potential seasonal bias

**Figure 7:**
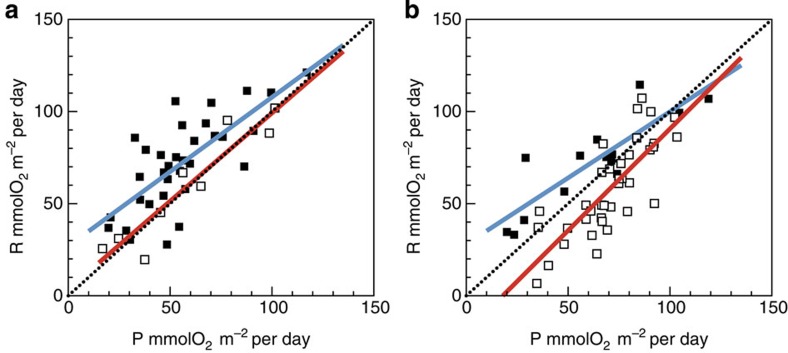
Seasonality of P:R relationships. Relationship between the integrated rates of photosynthesis (P) and respiration (R) in the NAST (filled squares) and SATL (open squares) in (**a**) autumn and (**b**) spring. The blue lines are the regression lines for the NAST data: *R*=0.80P+26.9, *R*^2^=0.54, *n*=33 (autumn); *R*=0.72P+28.01, *R*^2^=0.74, *n*=14 (spring). The red lines are the regression lines for the SATL data: *R*=0.96P+3.56; *R*^2^=0.87; *n*=9 (autumn); *R*=1.10P−19.79, *R*^2^=0.62, *n*=37 (spring). The dotted lines indicate the 1:1 line.

Most AMT cruises took place in boreal autumn, with only AMT12, 14 and 16 in spring. This implies that the seasonality of plankton metabolism in the gyres might bias our conclusions on regional variability. Seasonal differences in P, R and NCP in the NAST and SATL (Table 2) concur with a previous analysis of a partially overlapping data set^24^ (note that gyre boundaries were identified in Gist *et al*.^24^ from salinity/density fronts and surface chlorophyll rather than the depth of the thermocline and DCM). To assess a potential seasonal bias, we have separated the entire AMT11–22 data set into astronomical season and repeated the analysis. Geographic patterns of P, R and NCP remain unaltered irrespective of the season, except for the fact that autumn NCP in the S gyre is not different from metabolic balance, which nonetheless sustains the significant difference between the N and S gyres (Table 2). Moreover, the slope of the P:R relationship in the SATL remains 1 in both spring and autumn, differing from the <1 slopes of the N gyre relationships (Fig. 7). Although further empirical evidence is necessary to define the seasonality of plankton metabolism in the Atlantic gyres, our generalized analysis of regional differences appears unbiased.

## Discussion

The debate about the metabolic state of the oligotrophic ocean has evolved into a polarized dispute between two contrasting positions that are very difficult to reconcile (that is, the oligotrophic ocean is heterotrophic or it is autotrophic) and require substantial unaccounted for allochthonous inputs of either organic or inorganic nutrients^10^,^11^. Both positions arise from the same fundamental view that ‘the oligotrophic ocean' is a single steady-state ecosystem whose balance of trophic processes is meaningful across scales. This perception is evident in the very discussion about its ‘metabolic state', a property of either organisms or ecosystems whose calculation requires knowledge of the spatial and temporal scales of key trophic processes and connections in the system (production, accumulation, transport, consumption and oxidation of organic matter)^25^. However, the definition of the oligotrophic ocean is solely based on the level of productivity, controlled by the degree of water column stratification that determines nutrient limitation for the phytoplankton. Moreover, the oligotrophic ocean includes distant regions whose trophic connections are difficult to determine. Hence, estimating a single metabolic state for the global oligotrophic ocean from scattered measurements of P and R assumes scale independency in its trophic dynamics, that is, rests on the important assumptions of functional unity and steady state.

Our findings indicate that both this logic and the empirical evidence sustaining the debate are flawed. The systematic prevalence of autotrophy in the oligotrophic SATL challenges the generally accepted view that net heterotrophy prevails in *in vitro* NCP measurements in the oligotrophic ocean^10^,^11^, and the conclusion that such a prevalence is due to systematic methodological biases^11^,^19^. Moreover, the difference in NCP between the NAST and SATL refutes the hypothesis that a single type of balance prevails throughout the oligotrophic ocean^10^,^11^. The oligotrophic ocean is neither auto- nor heterotrophic, but functionally diverse. The debate has partially resulted from the use of universal P:R relationships where the heterotrophic NAST-E is over-represented.

The prevalence of R over P in the NAST implies carbon and energy deficits that need to be compensated through anoxygenic primary production, inputs of allochthonous organic matter or a higher efficiency in the use of metabolic energy. The paucity of reducing substrates and feeble redox gradients in the epipelagic zone of the oligotrophic ocean constrain the efficiency of chemoautotrophy and anoxygenic photosynthesis, whose relative contributions to euphotic zone carbon fluxes are <1% compared to oxygenic photosynthesis^26^,^27^, that is, too small to explain the metabolic differences between the NAST and SATL. Photoheterotrophic prokaryotes can use both light and organic matter for energy but require organic molecules as sources of carbon and electrons. Although proteorhodopsin-based and aerobic anoxygenic phototrophic bacteria are abundant and active in the oligotrophic ocean^28^, the implications of photoheterotrophy for the metabolic balance in the upper ocean are unclear. On one hand, phototrophic energy production reduces the need for aerobic respiration to sustain the maintenance costs of heterotrophs. On the other hand, photoheterotrophy increases the survival, and on occasions the growth, of prokaryotes under very limiting conditions^29^, possibly by supporting the energetic costs of processes such as active transmembrane transport, production of ectoenzymes, breakdown of complex organic molecules and cell motility^30^, but also by light induced anaplerotic CO_2_ fixation, which may provide up to 18% of cellular carbon demand^29^. This augmented survival, biomass production and the ability for organic matter utilization would increase the potential for heterotrophic respiration in the oligotrophic gyres. The consequences of photoheterotrophy for the metabolic cycling of carbon and the competitive advantage of planktonic bacteria^28^,^29^,^30^ suggest an important, and yet unknown, role in energy and carbon balances in the oligotrophic ocean. However, it is difficult to infer the relationship between photoheterotrophy and the regional differences in R and NCP, and photoheterotrophy would not reduce the estimated deficit of organic carbon in the NAST *per se*.

Calculations of dissolved organic carbon import to the surface of the NAST are one to two orders of magnitude too small to support previous estimations of net heterotrophy^11^. However, the mean mixed layer (upper 50 m) *in vitro* NCP deficit in the NAST used in these calculations (−1 mmolC m^−3^ per day)^11^ is ∼10 times higher than the −0.12 mmolO_2_ m^−3^ per day mean epipelagic NCP (average 112 m depth) estimated from our data set (Table 1; see also Fig. 5). In addition, our data show a depth-dependent distribution of NCP through the epipelagic layer, with lower heterotrophy near the 1% light depth (∼100 m depth) and in the upper 25 m (∼33% incident PAR; Figs 3, 4, 5). Although we are reluctant to calculate annual balances from our data set, assuming a respiratory quotient of 0.8^11^ and a mean mixed layer of 50 m^11^, 365 times our mean NCP in the upper 50 m of the NAST (−0.07±0.03 mmolO_2_ m^−3^ per day) amounts to −1.1±0.4 molC m^−2^ per year, that is, less than twice the 0.7 molC m^−2^ per year dissolved organic carbon input estimated ‘with high uncertainty' because of the ill constrained inputs from the margins^14^. These same lateral inputs are also needed to balance phosphorous budgets in the NAST. The phytoplankton of the North and South Atlantic gyres is limited primarily by nitrogen availability^31^; however, a distinctive characteristic of the NAST is that the concentration of bioavailable phosphorous in the euphotic layer is the lowest of all the ocean gyres and limits phytoplankton production^13^. The similar rates of phytoplankton carbon fixation^32^ and P (Table 1) in the NAST and SATL hence concur with the similar euphotic zone concentrations of bioavailable nitrogen, but are at odds with the chronic phosphorous limitation in the NAST. The lateral input of dissolved organic phosphorous (DOP) from the shelf region of NW Africa into the gyre interior helps to balance this discrepancy^33^. DOP imported by a combination of gyre and eddy circulations is estimated to support up to 70% of the particle export over much of the gyre^33^, and should therefore impact on the balance between P and R. DOP may be directly utilized by the phytoplankton or recycled by heterotrophic bacteria, but the latter have been found to easily outcompete *Prochlorococcus* (the most abundant phytoplankter in the oligotrophic ocean) for DOP (ATP) uptake^34^. Estimating the metabolic impact of DOP import into the NAST requires resolving the variability of group-specific differences in DOP uptake and utilization, which might also interact with the photoheterotrophic enhancement of dissolved organic matter bioavailability, and with an anticipated increase in the open ocean phosphorous limitation with global warming^35^. A mechanistic understanding of the regional differences in community metabolism requires not only solving the spatial and temporal variation of NCP and relative inputs of allochthonous inorganic and organic nutrients within the gyres, but also the study of the multiple biochemical strategies for nutrient stress^36^ and energy and carbon acquisition across spatial and temporal scales^37^.

Our results show that R heterogeneity in the oligotrophic ocean is important for NCP variability and prediction, and that global metabolic balances in the oligotrophic ocean represent the average of different metabolic states, rather than the metabolic state of a single global biome. While the potential of P:R relationships for NCP scaling remains, extrapolation should be specific to provinces showing a coherent trophic functioning^38^. The different geographic extent of these provinces requires spatially explicit approaches, that is, a trophic biogeographic partitioning of the ocean based on the spatial and temporal variability not only of P but also of R^23^,^38^. This is a difficult task because only the substantial P data set and reliable predictive algorithms^39^ allow for comprehensive regional and seasonal depictions^39^,^40^,^41^, which is not enough to reveal the trophic diversity of the ocean. Besides, the scale dependency of ecosystem metabolism along the temporal axis is arguably as important for the highly dynamic, non-steady-state planktonic ecosystem, and particularly relevant in the context of anthropogenic change^42^. Although R is a slow variable compared with P, its temporal variability could be important for prediction of trophic dynamics over scales significant to environmental change^43^.

The estimation of the metabolic balance of the oligotrophic ocean continues to be an important and urgent challenge for ocean biogeochemistry. Integrative *in situ* approaches are invaluable for constraining metabolic balances over long and large scales. However, only *in vitro* or other instantaneous methods may resolve the spatial heterogeneity and temporal dynamics of ecosystem processes, which is a prerequisite for prediction. Our findings call for an effort to improve the scope and resolution of R and NCP measurements in the ocean. While we are far from a mechanistic understanding of the variability of plankton trophic functioning, the scale difference between *in situ* and *in vitro* methods provides unique opportunities to derive and test system-dependent empirical models for NCP extrapolation. A better appreciation of the scaling of metabolic processes to biogeochemical fluxes is a priority to improve our prediction of the ocean's interaction with the changing climate.

## Methods

### Sampling

Ten latitudinal (∼50° N–45° S) transects of ∼70 stations across the Atlantic Ocean were conducted from 2000 to 2012 on RRS James Clark Ross, RRS Discovery and RRS James Cook between the United Kingdom and South America (Fig. 1). All the cruises except AMT17 and AMT18 included measurements of metabolism in both the North and South gyres (NAST and SATL). AMT cruises 11, 13, 15 and 17–22 took place in boreal autumn, while AMT12, 14 and 16 were in boreal spring. Details of sampling, incubation and analytical procedures for each cruise, including the complete cruise reports, may be found at http://www.amt-uk.org/ and Robinson *et al*.^44^

### Chlorophyll *a* concentration

During each cruise, vertical profiles of temperature and fluorescence were obtained twice daily (at ∼2 h before dawn and at solar noon) to a depth of ∼300 m with SeaBird and WETLabs Wet star sensors fitted to a SeaBird 9/11 plus CTD system. Chlorophyll *a* concentration was derived from calibrated fluorescence readings. A rosette of 24 × 20 l Niskin bottles fitted with the CTD system was used to collect seawater samples from six to nine depths (including light depths sampled for photosynthesis and respiration incubations—see below) to calibrate the CTD fluorometer following Welschmeyer^45^. Samples of 250 ml were filtered through 47-mm 0.2-μm polycarbonate filters. The filters were placed in a vial with 10 ml 90% acetone and left in a freezer for 24 h. The samples were then analysed on a precalibrated Turner Designs Trilogy fluorometer with a non-acidified chl module (CHL NA #046) fitted.

### Plankton metabolism

Gross photosynthesis (P), dark community respiration (R) and NCP were determined from *in vitro* changes in dissolved O_2_ after 24-h light and dark bottle incubations. Although *in situ* incubations would be preferred to deck incubations, they were prevented by cruise logistics (AMT cruises are cruises of opportunity where stations last ∼2 h and are separated by ∼350 km (∼13,500 km transect sampled over 42 days). Seawater was sampled daily, ∼2 h before dawn, from three to six depths (median 5) using the rosette of 24 × 20 l Niskin bottles. The total number of suitable stations ranges from 8 (AMT17) to 37 (AMT22) (mean of 19). Water was collected into opaque polypropylene aspirators from 4 (AMT12), 5 (AMT11,14,15,16,17) or 6 (AMT13,18,21,22) light depths covering the PAR range from ∼97 to 1% of surface irradiance. Actual light depths changed between stations and cruises, but typically included 97, 33, 14, 7 and 1% of surface irradiance. Irradiance levels were determined from light measurements made the previous day, and the assumption that the deep fluorescence maximum approximated the depth to which 1% of surface irradiance reached. The depth of the 1% light in the next noon PAR profile was always within 7% of our estimated depth. Twelve to 18 120-cm^3^, gravimetrically calibrated, borosilicate glass bottles were carefully filled from each aspirator bottle using silicon tubing, overflowing by >250 ml. From each depth, four to six replicate ‘zero-time' bottles were fixed immediately with Winkler reagents (1 ml of 3 M MnCl or MnSO_4_ and 1 ml of (8 M KOH+4 M KI) solutions) added separately with an automatic multipipette. Two further sets of four to six replicates were incubated for 24 h in surface water-cooled deck incubators or in temperature controlled water baths at *in situ* temperatures. These numbers of replicates are enough to obtain average coefficients of variation of the O_2_ concentration measurements in the zero, dark and light bottles <0.1%, while allowing the daily completion by one person of all the analyses corresponding to five to six depths. One set of replicates was incubated in the dark, the other set in the equivalent irradiance to that found at the *in situ* sampling depth using various combinations of neutral density and blue plastic filters. Owing to limitations of sample water volume and analysis time during AMT12 and 13, we assumed that plankton community structure was homogeneous within the surface 15 m and so incubated a set of replicates sampled from the 55% light depth at 97% of surface irradiance. Flow cytometry data from AMT13 confirmed that this was a reasonable assumption, since picoplankton cell abundance varied by less than 5% between the two light depths throughout the transect, and nanoplankton varied by 11% (ref. 24). Incubations always started at dawn, and during the hours of darkness the incubators were covered with opaque screens to prevent interference from the ship's deck lights. After the 24-h incubation period, the dark and light bottles were fixed with Winkler reagents. Dissolved oxygen concentration was determined with automated Winkler titration systems using potentiometric (Metrohm 716 DMS Titrino)^46^ or photometric^47^ end-point detection. For the potentiometric method, aliquots of fixed samples were delivered with a 50-ml overflow pipette. Fixing, storage and standardization procedures followed the recommendations by Grasshoff *et al*.^48^ Production and respiration rates were calculated from the difference between the means of the replicate light and dark incubated bottles and zero-time analyses: NCP=ΔO_2_ in light bottles (mean of [O_2_] in 24-h light—mean zero time [O_2_]); R=ΔO_2_ in dark bottles (mean zero time [O_2_]—mean [O_2_] in 24-h dark); P=NCP+R. The average s.e.m. of both the NCP and R rate measurements (which includes both experimental and biological variability) was 0.18 (*n*=875 and 876, respectively) mmolO_2_ m^−3^ per day. Integrated values were obtained by trapezoidal integration of the volumetric data down to the depth of 1% surface incident photosynthetically active irradiance. The s.d. of integrated P, R and NCP was calculated through propagation of the random error in the volumetric measurements. Samples were excluded (16 stations) when significant negative values of either P or R were measured, or when the variance of replicated measurements was anomalously high. The NAST and SATL P, R and NCP data are normality distributed (Lilliefors test, *α*=0.05).

The complete volumetric data set of P, R and NCP is available at http://www.uea.ac.uk/environmental-sciences/people/People/Faculty+and+Research+Fellow/robinsonc#research maintained by Carol Robinson. The complete data set of all the physical, chemical and biological variables from each cruise is available at BODC (http://www.bodc.ac.uk/projects/uk/amt). The conditions under which the data may be used are in line with the NERC Data Policy (http://www.nerc.ac.uk/research/sites/data/policy/), and will be explained following a request, before the delivery of data.

## Additional information

**How to cite this article:** Serret, P. *et al*. Both respiration and photosynthesis determine the scaling of plankton metabolism in the oligotrophic ocean. *Nat. Commun.* 6:6961 doi: 10.1038/ncomms7961 (2015).

## Figures and Tables

**Table 1:** Metabolic rates in the North and South Atlantic subtropical gyres.

	**NCP NAST**	**NCP SATL**	**P NAST**	**P SATL**	**R NAST**	**R SATL**
Integrated (±s.e.m.)	**−14.5±2.4**	**9.8±2.3**	**57.5±3.5**	**68.3±3.1**	**72.1±3.5**	**58.5±3.8**
95% CI for the mean	−19.3 to −9.8	5.0 to 14.6	50.84 to 64.17	61.6 to 75.1	64.8 to 79.4	51.2 to 65.9
Median	−14.5	11	54.8	68.5	73.4	54.8
Range	−60.5 to 20.6	−21.0 to 42.3	19.8 to 118.9	16.8 to 103.4	27.8 to 121.2	6.7 to 107.2
*n*	47	46	47	46	47	46
*P* value	0.0001		0.026		0.0001	
						
Volumetric (±s.e.m.)	**−0.12±0.02**	**0.06±0.02**	0.56±0.02	0.55±0.02	**0.68±0.03**	**0.48±0.02**
95% CI for the mean	−0.16 to −0.08	0.03 to 0.1	0.52 to 0.61	0.50 to 0.59	0.63 to 0.73	0.43 to 0.52
Median	−0.09	0.07	0.52	0.50	0.64	0.45
Range	−1.06 to 0.48	−1.30 to 0.75	0.00 to 2.38	0.00 to 1.58	0.00 to 2.75	0.00 to 2.03
*n*	202	233	202	233	204	237
*P* value	0.0001		0.57		0.0001	

CI, confidence interval; NCP, net community production; NAST, North Atlantic gyre; P, photosynthesis; R, respiration; SATL, South Atlantic gyre.

Mean (±s.e.), 95% confidence interval for the mean, median, range and number of observations of euphotic zone integrated (mmolO_2_ m^−2^ per day) and volumetric (mmolO_2_ m^−3^ per day) rates of NCP, P and R measured in the NAST and SATL provinces during AMT11–22 cruises. Numbers in bold indicate statistically significant differences (at *α*=0.05) between metabolic rates in the NAST and SATL. *P* value is the probability, assuming the null hypothesis, of the results of the Student's *t*-tests performed to compare the means of P, R and NCP rates in the NAST and SATL.

**Table 2:** Metabolic rates in spring and autumn in the Atlantic subtropical gyres.

	**NCP NAST**	**NCP SATL**	**P NAST**	**P SATL**	**R NAST**	**R SATL**
Astronomical spring (±s.e.m.)	**−10.4±2.2**	**12.3±2.4**	61.7±4.2	70.8±2.6	72.4±3.5	58.4±3.7
95% CI for the mean	−18.9 to −1.9	7.1 to 17.6	50.1 to 73.3	63.6 to 77.9	56.0 to 85.8	50.13 to 66.6
Median	−9.12	14	66.6	70.4	75.1	50.1
Range	−45.9 to 12.6	−21.0 to 42.3	20.0 to 119.0	34.7 to 103.4	33.1 to 115.0	6.68 to 107.2
*n*	14	37	14	37	14	37
*P* value	0.0001		0.19		0.079	
						
Astronomical autumn (±s.e.m.)	**−16.3±2.5**	**−0.73±1.5**	55.7±3.2	58.2±4.5	72.0±3.5	59.2±4.6
95% CI for the mean	−22.1 to −10.6	−11.7 to 10.3	47.2 to 64.3	41.9 to 74.6	62.8 to 81.2	41.6 to 76.8
Median	−16.9	−0.3	52.9	56.3	71.7	59.5
Range	−60.5 to 20.6	−16.7 to 14.4	19.8 to 117.0	16.8 to 101.0	27.8 to 121.2	19.6 to 102.0
*n*	33	9	33	9	33	9
*P* value	0.015		0.79		0.20	

CI, confidence interval; NCP, net community production; NAST, North Atlantic gyre; P, photosynthesis; R, respiration; SATL, South Atlantic gyre.

Mean (±s.e.), 95% confidence interval for the mean, median, range and number of observations of integrated rates of NCP, P and R (mmolO_2_ m^−2^ per day) measured in spring and autumn in the NAST and SATL provinces during AMT11–22 cruises. Numbers in bold indicate statistically significant differences (at *α*=0.05) between metabolic rates in the NAST and SATL. *P* value is the probability, assuming the null hypothesis, of the results of the Student's *t*-tests performed to compare the means of P, R and NCP rates in the NAST and SATL.

## References

[b1] Falkowski P. G. The role of phytoplankton photosynthesis in global biogeochemical cycles. Photosynth. Res. 39, 235–258 (1994). 24311124 10.1007/BF00014586

[b2] Falkowski P. G., Fenchel T. & Delong E. F. The microbial engines that drive Earth's biogeochemical cycles. Science 320, 1034–1039 (2008). 18497287 10.1126/science.1153213

[b3] Ducklow H. W. & Doney S. C. What is the metabolic state of the oligotrophic ocean? A debate. Ann. Rev. Mar. Sci. 5, 525–533 (2013). 10.1146/annurev-marine-121211-172331 22809191

[b4] Emerson S. . Experimental determination of organic carbon flux from open-ocean surface waters. Nature 389, 951–954 (1997).

[b5] Neuer S. . Differences in the biological carbon pump at three subtropical ocean sites. Geophys. Res. Lett. 29, 321–324 (2002).

[b6] Christian J. R. Biogeochemical cycling in the oligotrophic ocean: Redfield and non-Redfield models. Limnol. Oceanogr. 50, 646–657 (2005).

[b7] Polovina J. J., Howell E. A. & Abecassis M. Ocean's least productive waters are expanding. Geophys. Res. Lett. 35, L03618 (2008).

[b8] Bidigare R. R. . Subtropical ocean ecosystem structure changes forced by North Pacific climate variations. J. Plankton Res. 31, 1131–1139 (2009).

[b9] del Giorgio P. A., Cole J. J. & Cimbleris A. Respiration rates in bacteria exceed phytoplankton production in unproductive aquatic systems. Nature 385, 148–151 (1997).

[b10] Duarte C. M., Regaudie-de-Gioux A., Arrieta J. M., Delgado-Huertas A. & Agustí S. The oligotrophic ocean is heterotrophic. Ann. Rev. Mar. Sci. 5, 551–569 (2013). 10.1146/annurev-marine-121211-172337 22809189

[b11] Williams P.J.le. B., Quay P. D., Westberry T. K. & Behrenfeld M. J. The oligotrophic ocean is autotrophic. Ann. Rev. Mar. Sci. 5, 535–549 (2013). 10.1146/annurev-marine-121211-172335 22809190

[b12] Emerson S., Stump C. & Nicholson D. Net biological oxygen production in the ocean: remote *in situ* measurements of O_2_ and N_2_ in surface waters. Global Biogeochem. Cy. 22, GB3023 (2008).

[b13] Lipschultz F., Bates N. R., Carlson C. A. & Hansell D. A. New production in the Sargasso Sea: History and current status. Global Biogeochem. Cy. 16, 1–17 (2002).

[b14] Hansell D. A., Ducklow H. W., Macdonald A. M. & Baringer M. O. Metabolic poise in the North Atlantic Ocean diagnosed from organic matter transports. Limnol. Oceanogr. 49, 1084–1094 (2004).

[b15] Kähler P., Oschlies A., Dietze H. & Koeve W. Oxygen, carbon, and nutrients in the oligotrophic eastern subtropical North Atlantic. Biogeosciences 7, 1143–1156 (2010).

[b16] Williams P. J.le. B. The balance of plankton respiration and photosynthesis in the open oceans. Nature 394, 55–57 (1998).

[b17] Duarte C. M. & Agustí S. The CO_2_ balance of unproductive aquatic ecosystems. Science 281, 234–236 (1998). 9657712 10.1126/science.281.5374.234

[b18] Regaudie-de-Gioux A. & Duarte C. M. Global patterns in oceanic planktonic metabolism. Limnol. Oceanogr. 58, 977–986 (2013).

[b19] Westberry T. B., Williams P. J.le. B. & Behrenfeld M. J. Global net community production and the putative net heterotrophy of the oligotrophic oceans. Global Biogeochem. Cy. 26, GB4019 (2012).

[b20] Arístegui J. & Harrison W. G. Decoupling of primary production and community respiration in the ocean: implications for regional carbon studies. Aquat. Microb. Ecol. 29, 199–209 (2002).

[b21] Karl D. M., Laws E. A., Morris P., Williams P.J.le.B. & Emerson S. Metabolic balance of the open sea. Nature 426, 32 (2003). 14603308 10.1038/426032a

[b22] Williams P. J.le. B., Morris P. & Karl D. M. Net community production and metabolic balance at the oligotrophic ocean site, station ALOHA. Deep-Sea Res. I 51, 1563–1578 (2004).

[b23] Serret P., Fernández E. & Robinson C. Biogeographic differences in the net ecosystem metabolism of the open ocean. Ecology 83, 3225–3234 (2002).

[b24] Gist N., Serret P., Woodward E. M. S., Chamberlain K. & Robinson C. Seasonal and spatial variability in plankton production and respiration in the Subtropical Gyres of the Atlantic Ocean. Deep-Sea Res. II 56, 931–940 (2009).

[b25] Smith S. V. & Hollibaugh J. T. Annual cycle and interannual variability of ecosystem metabolism in a temperate climate embayment. Ecol. Monogr. 67, 509–533 (1997).

[b26] Middelburg J. J. Chemoautotrophy in the ocean. Geophys. Res. Lett. 38, L24604 (2011).

[b27] Goericke R. Bacteriochlorophyll a in the ocean: is anoxygenic bacterial photosynthesis important? Limnol. Oceanogr. 47, 290–295 (2002).

[b28] Kirchman D. L. & Hanson T. E. Bioenergetics of photoheterotrophic bacteria in the oceans. Environ. Microbiol. Rep. 5, 188–199 (2013). 23584962 10.1111/j.1758-2229.2012.00367.x

[b29] Palovaara J. . Stimulation of growth by proteorhodopsin phototrophy involves regulation of central metabolic pathways in marine planktonic bacteria. Proc. Natl Acad. Sci. USA 111, E3650–E3658 (2014). 25136122 10.1073/pnas.1402617111 PMC4156726

[b30] Koblížek J. in Microbial Carbon Pump in the Ocean (eds Jiao N., Azam F., Sanders S. 49–51Science/AAAS (2011).

[b31] Moore C. M. . Processes and patterns of oceanic nutrient limitation. Nat. Geosci. 6, 701–710 (2013).

[b32] Poulton A. J. . Phytoplankton carbon fixation, chlorophyll-biomass and diagnostic pigments in the Atlantic Ocean. Deep-Sea Res. II 53, 1593–1610 (2006).

[b33] Reynolds S., Mahaffey C., Roussenov V. & Williams R. G. Evidence for production and lateral transport of dissolved organic phosphorus in the eastern subtropical North Atlantic. Global Biogeochem. Cy. 28, 805–824 (2014).

[b34] Björkman K., Duhamel S. & Karl D. M. Microbial group specific uptake kinetics of inorganic phosphate and adenosine-5′-triphosphate (ATP) in the North Pacific Subtropical Gyre. Front. Microbiol. 3, 1–17 (2012). 22701449 10.3389/fmicb.2012.00189 PMC3371651

[b35] Karl D. M., Bidigare R. R. & Letelier R. M. Long-term changes in plankton community structure and productivity in the North Pacific subtropical gyre: the domain shift hypothesis. Deep-Sea Res. II 48, 1449–1470 (2001).

[b36] Saito M. A. . Multiple nutrient stresses at intersecting Pacific Ocean biomes detected by protein biomarkers. Science 345, 1173–1177 (2014). 25190794 10.1126/science.1256450

[b37] Karl D. M. Microbial oceanography: paradigms, processes and promise. Nat. Rev. Microbiol. 5, 759–769 (2007). 17853905 10.1038/nrmicro1749

[b38] Serret P. . Predicting plankton net community production in the Atlantic Ocean. Deep-Sea Res. II 56, 941–953 (2009).

[b39] Carr M. E. . A comparison of global estimates of marine primary production from ocean color. Deep-Sea Res. II 53, 741–770 (2006).

[b40] Chavez F. P., Messié M. & Pennington J. T. Marine primary production in relation to climate variability and change. Ann. Rev. Mar. Sci. 3, 227–260 (2011). 10.1146/annurev.marine.010908.163917 21329205

[b41] Longhurst A. Ecological Geography of the Sea 2, Academic Press (2006).

[b42] Wolkovich E. M., Cook B. I., McLauchlan K. K. & Davies T. J. Temporal ecology in the Anthropocene. Ecol. Lett. 17, 1365–1379 (2014). 25199649 10.1111/ele.12353

[b43] Carpenter S. R. & Turner M. G. Hares and tortoises: interactions of fast and slow variables in ecosystems. Ecosystems 3, 495–497 (2001).

[b44] Robinson C. . The Atlantic Meridional Transect (AMT) Programme: a contextual view 1995-2005. Deep Sea Res. II 53, 1485–1515 (2006).

[b45] Welschmeyer N. A. Fluorometric analysis of chlorophyll-a in the presence of chlorophyll-b and phaeopigments. Limnol. Oceanogr. 39, 1985–1992 (1994).

[b46] Oudot C., Gerard R., Morin P. & Gningue I. Precise shipboard determination of dissolved oxygen (Winkler procedure) for productivity studies with a commercial system. Limnol. Oceanogr. 33, 146–150 (1988).

[b47] Williams P. J.le. B. & Jenkinson N. W. A transportable micro-processor con- trolled precise Winkler titration suitable for field station and shipboard use. Limnol. Oceanogr. 27, 576–584 (1982).

[b48] (eds Grasshoff K., Ehrhardt M., Kremling K. Methods of Seawater Analysis 2nd edn 419Verlag Chemie (1983).

