# Corrigendum: Both respiration and photosynthesis determine the scaling of plankton metabolism in the oligotrophic ocean

**DOI:** 10.1038/ncomms12007

**Published:** 2016-06-15

**Authors:** Pablo Serret, Carol Robinson, María Aranguren-Gassis, Enma Elena García-Martín, Niki Gist, Vassilis Kitidis, José Lozano, John Stephens, Carolyn Harris, Rob Thomas

*Nature Communications* 6:6961 doi: 10.1038/ncomms7961 (2015); Published 04242015; Updated 2016

The original version of this Article failed to fully credit the use of the Ocean Data View software in figure 3, which appears below:

Schlitzer, R., Ocean Data View, http://odv.awi.de, 2016.

